# Transcriptome and expression profiling analysis revealed changes of multiple signaling pathways involved in immunity in the large yellow croaker during *Aeromonas hydrophila *infection

**DOI:** 10.1186/1471-2164-11-506

**Published:** 2010-09-22

**Authors:** Yinnan Mu, Feng Ding, Peng Cui, Jingqun Ao, Songnian Hu, Xinhua Chen

**Affiliations:** 1 Key Laboratory of Marine Biogenetic Resources, Third Institute of Oceanography, State Oceanic Administration, Xiamen 361005, China; 2 The CAS Key Laboratory of Genome Sciences and Information, Beijing Institute of Genomics, Chinese Academy of Sciences, Beijing 100029, China

## Abstract

**Background:**

The large yellow croaker (*Pseudosciaena crocea*) is an economically important marine fish in China suffering from severe outbreaks of infectious disease caused by marine bacteria such as *Aeromonas hydrophila *(*A. hydrophila*), resulting in great economic losses. However, the mechanisms involved in the immune response of this fish to bacterial infection are not fully understood. To understand the molecular mechanisms underlying the immune response to such pathogenic bacteria, we used high-throughput deep sequencing technology to investigate the transcriptome and comparative expression profiles of the large yellow croaker infected with *A. hydrophila*.

**Results:**

A total of 13,611,340 reads were obtained and assembled into 26,313 scaffolds in transcriptional responses of the *A. hydrophila*-infected large yellow croaker. Via annotation to the NCBI database, we obtained 8216 identified unigenes. In total, 5590 (68%) unigenes were classified into Gene Ontology, and 3094 unigenes were found in 20 KEGG categories. These genes included representatives from almost all functional categories. By using Solexa/Illumina's DeepSAGE, 1996 differentially expressed genes (P value < 0.05) were detected in comparative analysis of the expression profiles between *A. hydrophila*-infected fish and control fish, including 727 remarkably upregulated genes and 489 remarkably downregulated genes. Dramatic differences were observed in genes involved in the inflammatory response. Bacterial infection affected the gene expression of many components of signaling cascades, including the Toll-like receptor, JAK-STAT, and MAPK pathways. Genes encoding factors involved in T cell receptor (TCR) signaling were also revealed to be regulated by infection in these fish.

**Conclusion:**

Based on our results, we conclude that the inflammatory response may play an important role in the early stages of infection. The signaling cascades such as the Toll-like receptor, JAK-STAT, and MAPK pathways are regulated by *A. hydrophila *infection. Interestingly, genes encoding factors involved in TCR signaling were revealed to be downregulated by infection, indicating that TCR signaling was suppressed at this early period. These results revealed changes of multiple signaling pathways involved in immunity during *A. hydrophila *infection, which will facilitate our comprehensive understanding of the mechanisms involved in the immune response to bacterial infection in the large yellow croaker.

## Background

The large yellow croaker (*Pseudosciaena crocea*) is an economically important marine fish in China, with an annual yield that exceeds any other single netcage-farmed marine species. However, recent rapid development of the large yellow croaker farming industry has led to increasingly severe outbreaks of infectious disease caused by marine bacteria such as *Aeromonas hydrophila *(*A. hydrophila*), resulting in great economic losses [1]. However, little is known about the molecular mechanisms underlying the immune response to such pathogenic bacteria in this fish species, thereby hindering the establishment of effective measures in disease control [2].

Cellular identity and function are determined by the transcriptome or the complete repertoire of expressed RNA transcripts. Transcriptome profiling is a powerful method for assessing the relative importance of gene products in any chosen cell, tissue, organism, or condition. During the last few years, several methods have been used to study the fish transcriptome, including ESTs in channel catfish [3], Atlantic salmon [4], and orange-spotted grouper [5], as well as microarrays in adult zebrafish [6], rainbow trout [7], blue catfish [8], medaka, and Xiphophorus maculates [9]. However, microarrays are limited by background and cross-hybridization problems and only measure the relative abundance of transcripts. Moreover, only predefined sequences are detected [10]. EST sequencing techniques have limitations in the depth of the transcriptome that can be sampled [11].

Recent rapid developments of high-throughput deep sequencing technologies have provided an unprecedented increase in transcriptome data [12]. These next-generation sequencing platforms, such as the Solexa/Illumina Genome Analyzer and ABI/SOLiD Gene Sequencer, can sequence in parallel massive amounts of DNA molecules derived directly from mRNA, producing millions or even billions of high-quality short reads [13,14]. DeepSAGE is a tag sequencing method on the Illumina high-throughput sequencing platform that is analogous to LongSAGE [15,16]. Compared to LongSAGE, DeepSAGE provides much more sensitive and cost-efficient gene expression profiling [15,16]. By using this technology, some progress has recently been made in the characterization of the immune mechanisms and pathways in zebrafish [17]. Nevertheless, there are still important gaps in the knowledge of numerous immune mechanisms, and the available information varies according to the fish species [18].

Here, the large yellow croaker was used as a model to investigate the host response to *A. hydrophila *infection. First, a transcriptome library was constructed from spleen isolated from *A. hydrophila*-infected fish. Deep sequencing was accomplished using the Solexa/Illumina sequencing technology. Using the SOAP *de novo *transcriptome assembly software, we ultimately obtained a transcriptome database containing 8216 identified unigenes. Quantitative gene expression analysis was performed using DeepSAGE technology. Tags identified from normal and bacteria-infected fish were mapped to the transcriptome database above for comparative analysis. A reference set of significantly upregulated and downregulated immune-related genes was compiled.

## Results

### Transcriptome profile of the large yellow croaker (*Pseudosciaena crocea)*

**Figure 1:**
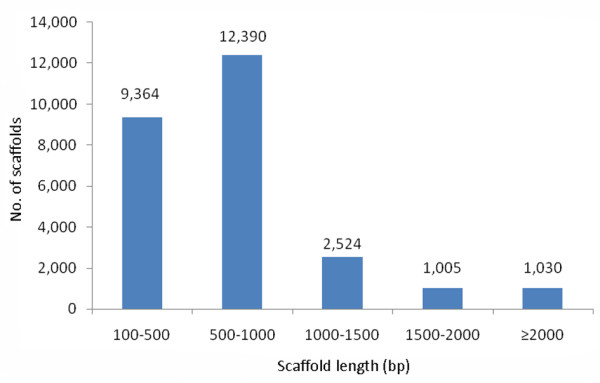
**Length statistics of scaffolds obtained from the large yellow croaker transcriptome library**. The length distributions of the transcriptome library. Sequences with lengths of 500-1000 bp were most abundant, making up 47% of the scaffolds.

To better understand the molecular mechanisms of the large yellow croaker immune system, we constructed a Solexa cDNA library from the spleen of fish infected with *A. hydrophila*. High-throughput paired-end sequencing yielded a total of 13,611,340 reads. Of these, 901,200 reads containing more than five consecutive bases with a quality < 13 were removed. The remaining 12,710,140 high-quality reads were assembled into 26,313 scaffolds by using the SOAP *de novo *software, with a maximum scaffold length of 7585 bp. The length statistics of all scaffolds are presented in Figure 1.

Scaffold annotation was achieved through BLASTN similarity searches against the zebrafish RefSeq mRNA database (version danRer5). This analysis revealed that 10,502 of the 26,313 scaffolds (40%) shared homology with zebrafish genes when a cutoff E-value of 1e-05 was used. Scaffolds were clustered if two or more query sequences were annotated to the same zebrafish gene. Ultimately, 5715 unigenes were obtained. Scaffolds that did not display any similarity to zebrafish genes were further searched against the nonredundant (nr) database, and 2501 unigenes were obtained after clustering. In total, 8216 unigenes were identified in the transcriptome of the large yellow croaker (Additional file 1, Table S1). The remaining 13,102 scaffolds failed to match proteins in the nr database and therefore represented potentially novel genes.

Gene ontology (GO) analysis of these genes was performed using the web-based Database for Annotation, Visualization, and Integrated Discovery (DAVID) [19,20]. Among the 8216 unigenes, DAVID had functional annotation for 5590 genes. The DAVID functional annotation analysis for GO (level 2) is summarized in Table 1. Sequences with GO terms corresponding to the "cellular component" group fell into 14 subcategories, "molecular function" into 16 subcategories, and "biological process" into 31 subcategories. The largest subcategory found in the "cellular component" group was 'cell part,' which comprised 98.8% of the genes in this subcategory. In the "molecular function" and "biological process" categories, "nucleotide binding" and "primary metabolic process" were the most abundant GO terms, making up 22.4% and 50.2% of each subcategory, respectively.

**Table 1:** GO function annotation results of 8216 unigenes

Term	GO ID	Description	**Gene No**.	%*	P Value
CC_2	GO:0044464	cell part	2650	98.84	0.000013
CC_2	GO:0005622	intracellular	2041	76.13	0.000000
CC_2	GO:0044424	intracellular part	1707	63.67	0.000000
CC_2	GO:0043229	intracellular organelle	1351	50.39	0.000000
CC_2	GO:0043227	membrane-bounded organelle	1103	41.14	0.000000
CC_2	GO:0044446	intracellular organelle part	466	17.38	0.000000
CC_2	GO:0044422	organelle part	466	17.38	0.000000
CC_2	GO:0043234	protein complex	410	15.29	0.000000
CC_2	GO:0043228	non-membrane-bounded organelle	366	13.65	0.000000
CC_2	GO:0030529	ribonucleoprotein complex	210	7.83	0.000000
CC_2	GO:0031090	organelle membrane	137	5.11	0.001153
CC_2	GO:0043233	organelle lumen	135	5.04	0.000000
CC_2	GO:0031967	organelle envelope	98	3.66	0.000398
CC_2	GO:0012505	endomembrane system	86	3.21	0.021749
MF_2	GO:0000166	nucleotide binding	827	22.39	0.000000
MF_2	GO:0016787	hydrolase activity	674	18.25	0.000000
MF_2	GO:0016740	transferase activity	606	16.4	0.000001
MF_2	GO:0001882	nucleoside binding	506	13.7	0.000000
MF_2	GO:0016874	ligase activity	122	3.3	0.000001
MF_2	GO:0003735	structural constituent of ribosome	118	3.19	0.000000
MF_2	GO:0048037	cofactor binding	96	2.6	0.001411
MF_2	GO:0060589	nucleoside-triphosphatase regulator activity	77	2.08	0.001763
MF_2	GO:0008135	translation factor activity, nucleic acid binding	60	1.62	0.000000
MF_2	GO:0016853	isomerase activity	58	1.57	0.000002
MF_2	GO:0003702	RNA polymerase II transcription factor activity	32	0.87	0.000002
MF_2	GO:0051540	metal cluster binding	24	0.65	0.026354
MF_2	GO:0008430	selenium binding	16	0.43	0.031351
MF_2	GO:0019825	oxygen binding	11	0.3	0.022460
MF_2	GO:0004601	peroxidase activity	9	0.24	0.072047
MF_2	GO:0008641	small protein activating enzyme activity	5	0.14	0.071494
BP_2	GO:0044238	primary metabolic process	1571	50.18	0.000000
BP_2	GO:0044237	cellular metabolic process	1466	46.82	0.000000
BP_2	GO:0043170	macromolecule metabolic process	1271	40.59	0.000000
BP_2	GO:0009058	biosynthetic process	637	20.34	0.000000
BP_2	GO:0006807	nitrogen compound metabolic process	608	19.42	0.000020
BP_2	GO:0051234	establishment of localization	533	17.02	0.051495
BP_2	GO:0006810	transport	528	16.86	0.055745
BP_2	GO:0009056	catabolic process	235	7.51	0.000000
BP_2	GO:0033036	macromolecule localization	214	6.83	0.000000
BP_2	GO:0045184	establishment of protein localization	178	5.69	0.000000
BP_2	GO:0006996	organelle organization	154	4.92	0.031676
BP_2	GO:0051641	cellular localization	139	4.44	0.000000
BP_2	GO:0051649	establishment of localization in cell	132	4.22	0.000000
BP_2	GO:0065008	regulation of biological quality	124	3.96	0.055589
BP_2	GO:0022607	cellular component assembly	107	3.42	0.001415
BP_2	GO:0042221	response to chemical stimulus	88	2.81	0.067111
BP_2	GO:0043933	macromolecular complex subunit organization	84	2.68	0.000005
BP_2	GO:0016192	vesicle-mediated transport	75	2.4	0.000639
BP_2	GO:0006066	alcohol metabolic process	64	2.04	0.022528
BP_2	GO:0019725	cellular homeostasis	63	2.01	0.000479
BP_2	GO:0070271	protein complex biogenesis	58	1.85	0.000005
BP_2	GO:0034621	cellular macromolecular complex subunit organization	58	1.85	0.003498
BP_2	GO:0002520	immune system development	54	1.72	0.000019
BP_2	GO:0051301	cell division	45	1.44	0.019598
BP_2	GO:0022613	ribonucleoprotein complex biogenesis	38	1.21	0.000011
BP_2	GO:0019637	organophosphate metabolic process	38	1.21	0.029496
BP_2	GO:0009893	positive regulation of metabolic process	37	1.18	0.060638
BP_2	GO:0006413	translational initiation	25	0.8	0.000041
BP_2	GO:0051236	establishment of RNA localization	15	0.48	0.017466
BP_2	GO:0042440	pigment metabolic process	14	0.45	0.004606
BP_2	GO:0044087	regulation of cellular component biogenesis	13	0.42	0.008675

*, indicates the percentage of genes in the specific subcategory from each of the three GO ontologies.

To identify the biological pathways that are active in the large yellow croaker, we mapped the 8216 genes to canonical signaling pathways found in the Kyoto Encyclopedia of Genes and Genomes (KEGG). A total of 3094 genes of the large yellow croaker transcriptome were mapped to KEGG, and 20 statistically remarkable categories (P value < 0.05) are listed in Table 2. The mitogen-activated protein kinase (MAPK) signaling pathway, neurotrophin signaling pathway, and chemokine signaling pathway were identified as statistically significant. In fact, 47 genes were found to be related to the MAPK pathway. Other major immune pathways, such as those mediated by the T cell receptor (TCR) and B cell receptor (BCR), were also statistically significant.

**Table 2:** Statistically significant KEGG classifications of large yellow croaker genes

Category	**Gene No**.	%*	P value
Huntington's disease	81	2.62	0.000000
Ribosome	70	2.26	0.000000
Pathways in cancer	70	2.26	0.000020
Oxidative phosphorylation	69	2.23	0.000000
Alzheimer's disease	67	2.17	0.000000
Parkinson's disease	62	2	0.000000
Ubiquitin mediated proteolysis	54	1.75	0.000000
Lysosome	54	1.75	0.000000
Purine metabolism	51	1.65	0.000192
MAPK signaling pathway	47	1.52	0.027690
Regulation of actin cytoskeleton	47	1.52	0.000330
Focal adhesion	43	1.39	0.000870
Pyrimidine metabolism	37	1.2	0.000079
Insulin signaling pathway	35	1.13	0.000092
Neurotrophin signaling pathway	35	1.13	0.000014
Chemokine signaling pathway	34	1.1	0.041450
Proteasome	32	1.03	0.000000
T cell receptor signaling pathway	29	0.94	0.000161
Leukocyte transendothelial migration	29	0.94	0.000788
B cell receptor signaling pathway	27	0.87	0.000001

*, indicates the percentage of genes in each pathway from 3094 genes mapped to KEGG.

### Global changes in gene expression upon *A. hydrophila *infection

To characterize the immune response of the large yellow croaker to bacterial infection, two DeepSAGE libraries were constructed using mRNA from spleens injected with *A. hydrophila *or 0.9% NaCl. After removal of the low-quality tags, adaptor tags, and one copynumber tag, a total of 4,841,402 and 5,395,715 clean tags were obtained from the two libraries with 100,107 and 108,572 unique nucleotide sequences, respectively (Additional file 2, Table S2). Subsequently, the tag sequences from the infected and control libraries were mapped to the transcriptome database described above. Approximately 50% of the tags matched sequences in the transcriptome, while 39% could be identified unequivocally by unique tag mapping (Additional file 3, Table S3). A total of 1996 differentially expressed genes (P value < 0.05) were found (Additional file 4, Table S4), including 1133 upregulated genes and 863 downregulated genes, in the spleen of fish infected with *A. hydrophila*. Particularly, 727 genes were upregulated at least 1.5-fold, including 208 genes that were unique to the infected library, while 489 genes were downregulated at least 1.5-fold, including 182 genes uniquely expressed in the control library.

To achieve a functional annotation of the infection-responsive genes, GO classifications were assigned to the 1996 differentially expressed genes by using DAVID (Additional file 5, Table S5). GO analysis indicated that bacterial infection up- and downregulated genes involved in immunity, transcription, translation regulations, and biological regulation.

**Figure 2:**
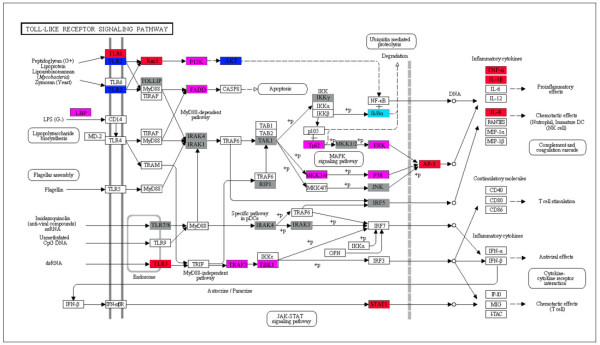
**Gene list involved in TLR pathway generated by KEGG**. Red indicates significantly increased expression; pink, not remarkably increased expression; blue, significantly decreased expression; cyan, not remarkably decreased expression; and gray, unchanged expression. White denotes genes that were not identified in the expression profile analysis.

Some significantly differentially expressed genes in expression profiles using GO classifications are shown in Table 3. The immune-related genes were enriched in GO terms "response to chemical stimulus" and "immune system development." Relative quantitative real-time PCR analysis was also performed to confirm the differentially expression genes. These genes were mapped to KEGG and found to be associated with the Toll-like receptor (TLR) signaling pathway (Figure 2). This group included TLR genes (e.g., *TLR1*, *TLR2*, *TLR3*, and *TLR22*), cytokine genes (e.g., *TNF-α*, *IL-1β*, and *IL-8*), and chemokine and chemokine receptor genes (e.g., *CCL-4*, *CCL-c25v*, *CCR-1*, *CCR-12.3*). Additionally, apoptosis-related genes, including *Casp9 *and *Fas*, as well as those involved in antioxidant activity such as *Prdx1*, *Prdx2*, *Gpx1b*, and *Gpx4b *were discovered. Genes involved in B cell and T cell development, such as *Blnk *and *CD3ζ/d*, were also found to be differentially expressed (Table 3). The B cell linker protein (Blnk), also known as SLP-65, is essential for normal B cell development by influencing the BCR signaling pathway [21]. The TCR/CD3ζ complex mediates antigen recognition and T cell stimulation, with CD3ζ/d playing a pivotal role in this process [22].

**Table 3:** Representative genes significantly differentially expressed after *A. hydrophila *infection

Gene Name	**Accession NO**.	Describe	Fold	P value
**Immunity related genes**				
TLR1	P79800	Toll-like receptor 1	18/0	0.000001
TLR2	NM_212812	Toll-like receptor 2	0/94	0
TLR3	BAD01045	Toll-like receptor 3	4/0	0
TLR22	NM_001128675	Toll-like receptor 22	-2.5	0
IL-1β	gb|AAP33156.1|	Interleukin-1β	+17.9	0
IL-8	XP_695462	Interleukin-8	+20.8	0.000007
IL-2rgb	NM_001123050	Interleukin 2 receptor, gamma b	+2.3	0.001956
IL-4r	NM_001013282	Interleukin 4 receptor	+1.4	0.000015
IL-6r	NM_001114318	Interleukin 6 receptor	+2.2	0.000023
CCL-4	CAO78735.1	CC chemokine ligand 4	+2.75	0
CCL-c25v	NM_001115103	Chemokine CCL-C25v	0/34	0
CCr-1	ref|NP_001028030.1	CC chemokine receptor type 1	+9.6	0
CCr-12.3	NM_001045027	CC chemokine receptor family-like	+4.4	0
Crlf-3	ref|NP_001167401.1	cytokine receptor-like factor 3	65/0	0
TNFaip8	NM_200332	TNF, alpha-induced protein 8-like protein 1	-2.2	0.043671
TNFsf10l2	NM_001002593	TNF superfamily, member 10 like 2	-1.3	0.00616
Jfmip1a	dbj|BAC10650.1|	MIP1alpha	-2.5	0.000505
Cklr	gb|AAP58737.1|	C-type lectin receptor	+1.7	0
Blnk	NM_212838	B-cell linker	-1.6	0
zgc:55347	NM_213522	Immunoglobulin binding protein 1	4/0	0.026366
Fcgr1	gb|ACN10126.1|	High affinity immunoglobulin gammaFc receptor I precursor	+2.6	0.049003
CD3g/d	ref|NP_001033072.1	CD3 gamma/delta	-1.9	0
Rad23b	NM_200564	RAD23 homolog B	+2.4	0.018151
Fas	ref|NP_001075464.	Fas	4/0	0.026366
Casp9	NM_001007404	caspase9 apoptosis-related cysteine protease	+5.8	0.000013
Was	emb|CAQ15295.1|	Wiskott-Aldrich syndrome	-3.3	0.017699
Tpsn	NM_130974	Tapasin	+2.3	0
Lipf	NM_213404	Lipf protein	+2.4	0
Hsp90a.1	NM_131328	Heat shock protein HSP 90-alpha 1	+1.2	0.048885
Gadd45al	NM_200576	Growth arrest and DNA-damage-inducible, beta	+1.7	0
Prdx1	NM_001013471	Peroxiredoxin 1	+5.8	0
Prdx2	NM_001002468	Peroxiredoxin 2	-1.5	0
Glrx5	NM_213021	Glutaredoxin 5	+1.3	0.015063
Gpx1b	NM_001004634	Glutathione peroxidase 1b	+1.4	0
Gpx4b	NM_001030070	Glutathione peroxidase 4b	-1.4	0
zgc:85657	NM_214749	Non-homologous end-joining factor 1	-1.4	0.02618
Mpx	NM_212779	Myeloid-specific peroxidase	-1.9	0.02238
Ube2nl	NM_200342	Ubiquitin-conjugating enzyme E2N-like	+2	0
**Transcription regulator activity**				
NF-kB2	NM_001001840	NF-kB, p49/p100	-1.7	0
NF-kBie	NM_001080089	NF-kB 2 inhibitor, epsilon	+1.7	0
Jak1	NM_131073	Janus kinase 1	+6.1	0.003023
Stat1	ref|NP_001117126.1|	STAT1 alpha	+3.9	0.027891
Jun	NM_199987	c-Jun	0/5	0.02144
Jund	NM_001128342	Jun D proto-oncogene	+1.5	0.012807
Xbp1	NM_131874	X-box binding protein 1	0/6	0.011301
Smad9	NM_001004014	Smad9	-3.6	0.031597
Slp-1	gb|AAC41262.1|	Transcription factor	0/4	0.040678
Srf	gb|AAH50480.1|	Srf protein	-1.5	0.006007
Tp53	NM_131327	Cellular tumor antigen p53	-1.2	0.031928
Cebpa	NM_131885	CCAAT/enhancer binding protein alpha	11/0	0.00014
Pdlim1	NM_001017870	PDZ and LIM domain 1	+2.1	0.000003
Ahr2b	ref|NP_001033052.1|	Aryl hydrocarbon receptor 2B	+2.3	0.00573
IRF	dbj|BAA83468.1|	interferon regulatory factor	+2.9	0
IRF4	NM_001122710	interferon regulatory factor 4	+1.2	0.040115
IRF9	NM_205710	interferon regulatory factor 9	+2.3	0
Max	NM_131220	Myc-associated factor X	+1.3	0.003228
Rargb	NM_001083310	Retinoic acid receptor gamma	13/0	0.000031
Ldb1a	NM_131313	LIM domain-binding protein 4	+1.6	0.0016
Cse1l	NM_201450	Chromosome segregation 1-like	-1.2	0.045136
Ppp1r10	NM_212568	protein phosphatase 1, regulatory subunit 10	-1.2	0.00723
Ppp1caa	NM_214811	protein phosphatase 1, catalytic subunit alpha	-3.9	0.007564
Gtf2h2	NM_201581	General transcription factor IIH, polypeptide 2	4/0	0.026366
Gtf2h3	NM_001002564	General transcription factor IIH, polypeptide 3	+2.5	0.000295
Gf2f2	NM_001017832	General transcription factor IIF, polypeptide 2	+1.4	0.021645
Gtf2e2	NM_212731	General transcription factor IIE, polypeptide 2, beta	+31.9	0

Limitations of all differentially expressed genes are based on P < 0.05. A P value < 0.05 indicated that the gene was significantly altered after bacterial challenge. The absolute value of "Fold" means the magnitude of up- or downregulation for each gene/homolog after bacterial challenge; "+" indicates upregulation, "-" indicates downregulation, and "0" indicates the gene was not found in one library. "Accession NO" is GenBank identifiers for the conformable reference sequences.

Many genes in the transcription regulation group were upregulated by *A. hydrophila *infection. This group includes genes encoding *NF-κB2*, *NF-κBie*, *IRF9*, *IRF11*, *Jund*, *Jak1*, *Stat1*, *Cebpa*, and *Cebpb *(Table 3). NF-κB is a transcription factor involved in regulating a large number of genes, especially cytokine genes [23]. *Jak1 *and *Stat1 *are components of the JAK-STAT signaling pathway. The remaining genes were represented by GO terms such as cellular component, binding, catalytic activity, structural molecular activity, and growth. These biological functions and pathways have not been associated directly with a particular immune-related event. Meanwhile, a number of uniquely expressed genes were hypothetical proteins, and future identification of these genes and their function may provide new insights into the immune response to *A. hydrophila *infection.

### GenMAPP analysis reveals genes involved in TCR and MAPK signaling

**Figure 3:**
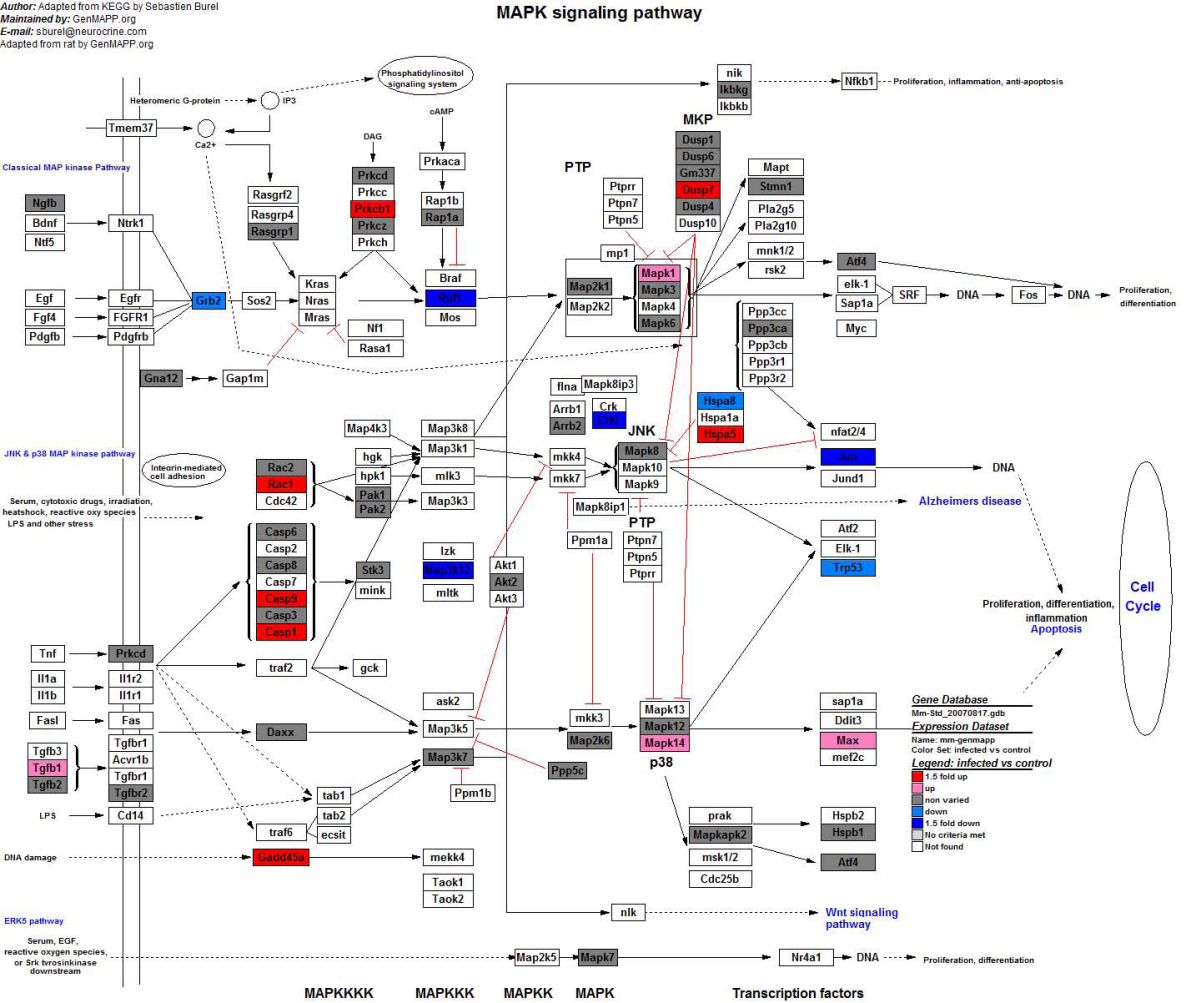
**The MAPK signaling pathways generated by GenMAPP**. The map-based pathway was made using the GenMAPP software package. A total of 4004 *Mus musculus *homologs were used to create the GenMAPP. *Mus musculus *homologs were identified by searching the 8216 unigenes against the zebrafish RefSeq data downloaded from the UCSC website and then the database of HomoloGene at the NCBI. Red indicates significantly increased expression; pink, not remarkably increased expression; blue, significantly decreased expression; cyan, not remarkably decreased expression; and gray, unchanged expression. White denotes genes that were not identified in the expression profile analysis.

**Figure 4:**
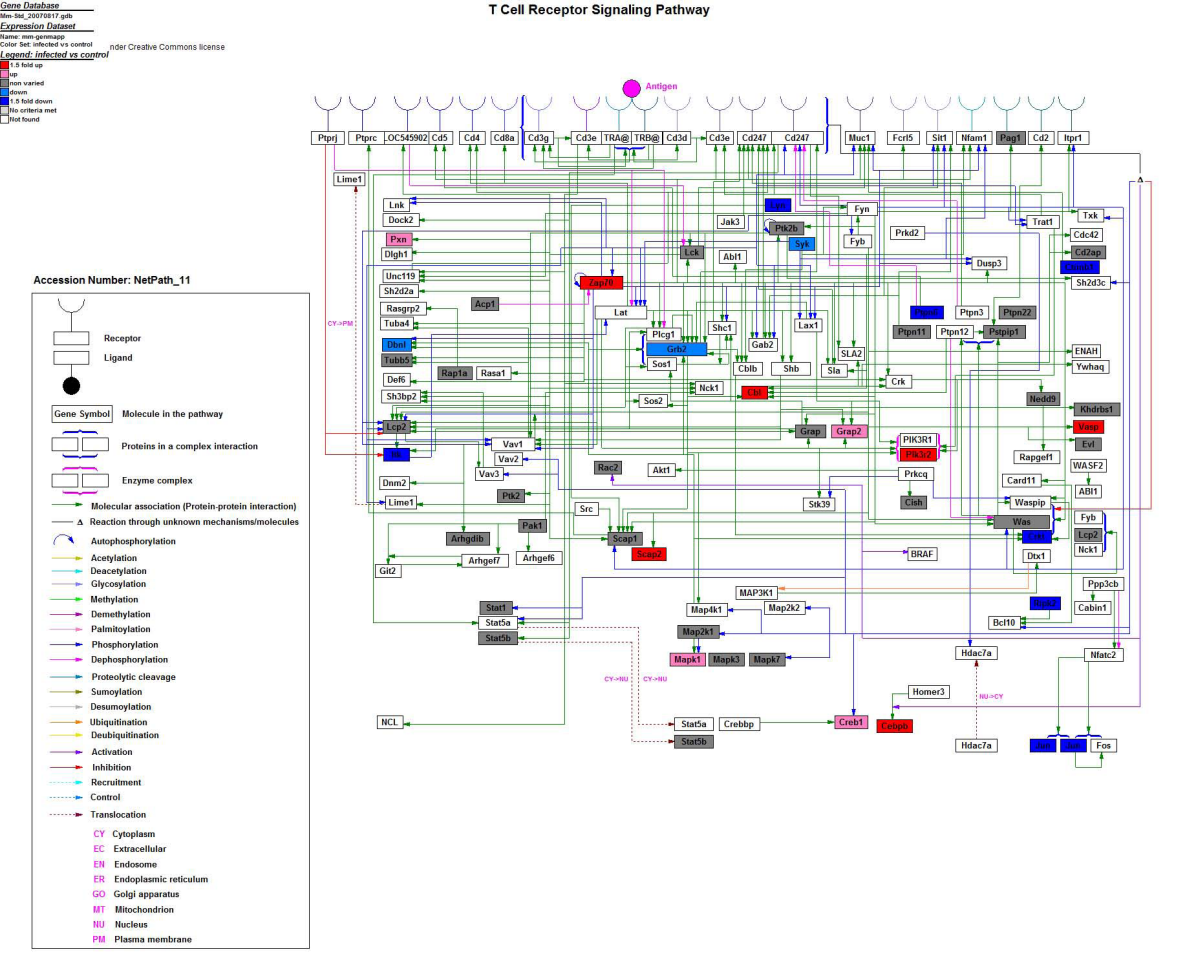
**The TCR signaling pathways generated by GenMAPP**. The map-based pathway was made using the GenMAPP software package. A total of 4004 *Mus musculus *homologs were used to create the GenMAPP. *Mus musculus *homologs were identified by searching the 8216 unigenes against the zebrafish RefSeq data downloaded from the UCSC website and then the database of HomoloGene at the NCBI. Red indicates significantly increased expression; pink, not remarkably increased expression; blue, significantly decreased expression; cyan, not remarkably decreased expression; and gray, unchanged expression. White denotes genes that were not identified in the expression profile analysis.

To further explore the immune response profiles induced by *A. hydrophila *infection to the level of a single pathway, we performed a map-based pathway analysis by using the GenMAPP software package http://www.genmapp.org/. In our study, 4004 *Mus musculus *homologs were used to create the GenMAPP. *Mus musculus *homologs were identified by searching the 8216 unigenes against the zebrafish RefSeq data downloaded from the UCSC website http://genome.ucsc.edu/ and then the database of HomoloGene at the NCBI http://www.ncbi.nlm.nih.gov. GenMAPP analysis was performed to identify genes involved in the MAPK pathway (Figure 3). In total, seven genes were identified as highly upregulated upon infection, *Casp9*, *Prkcb1*, *Hspa5*, *Radd45a*, *Dusp7*, *Rac1*, and *Casp1*. Contrarily, four genes were highly downregulated in response to *A. hydrophila *infection, *Map3k12*, *Crkl*, *Jun*, and *Raf1 *(Additional file 6, Table S6). We also used GenMAPP to analyze genes involved in TCR signaling. T cell activation, a key event in adaptive immunity, promotes a variety of signaling cascades that ultimately lead to cytokine production, cell survival, proliferation, and differentiation [24]. The resultant map (Figure 4) revealed eight remarkably downregulated genes (*Was*, *Lyn*, *Ptpn6*, *Ctnnb1*, *Itk*, *Crkl*, *Jun*, and *Ripk2*) and seven remarkably upregulated genes (*Khdrbs1*, *Scap2*, *Vasp*, *Pik3r2*, *Cebpb*, *Zap70*, and *Cbl*) involved in TCR signaling after *A. hydrophila *infection (Additional file 7, Table S7).

## Discussion

At present, molecular studies on the immune response to pathogens in the large yellow croaker are still rare. To increase our knowledge of host responses to bacterial infection, we firstly analyzed the transcriptome profile of the fish after *A. hydrophila *infection. Bioinformatic analysis of RNA-seq data should involve mapping of short reads to the genome [17]. However, genome and transcriptome resources for most vertebrate species have not yet been obtained, including the large yellow croaker. We analyzed the transcriptome of the large yellow croaker in advance and obtained a mass of sequence information. Then quantitative gene expression profile analysis was performed, and the tags were mapped to obtained transcriptome database. In the set of highly differentially expressed genes, a number of genes were reported to be involved in immunity and signal transduction, encoding receptors, cytokines, innate defense molecules, enzymes, signal transducers, transcription factors, and other functional proteins.

**Figure 5:**
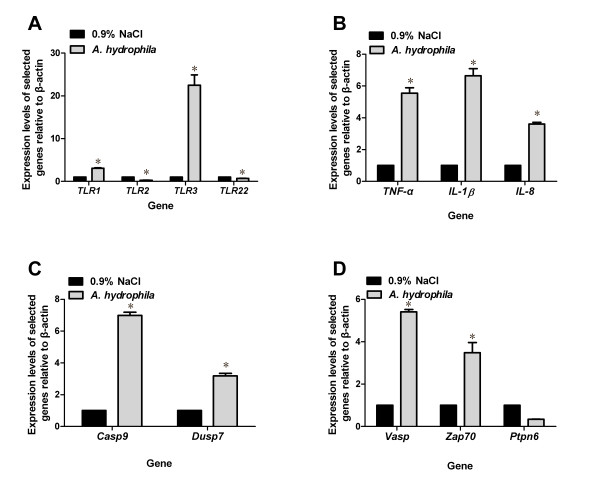
**The expression analysis of selected genes from the expression profile by relative quantitative real-time PCR**. Total RNA was extracted from spleens of fish infected with *A. hydrophila *or injected with 0.9% NaCl. Real-time PCR was used to validate gene expression changes in the TLR pathway **(A)**, cytokines **(B)**, the MAPK signaling pathway **(C)**, and the TCR signaling pathway **(D)**. Increases and decreases in relative levels of transcripts with respect to the control β-actin gene are shown. For each gene, the black bar indicates the gene expression ratio of fish injected with 0.9% NaCl and is defined as 1; the grey bar indicates the expression ratio of fish infected with *A. hydrophila*, with associated standard error bars. Statistical significance of the relative expression ratio is indicated (*, P < 0.01).

The innate immune system represents an efficient first line of defense against invading microbial pathogens. TLRs signal the presence of pathogens and elicit an innate immune response. This process has been reported in zebrafish infected with *Mycobacterium marinum *[25,26]. Our data revealed 35 genes involved in TLR cascades in the transcriptome of infected large yellow croaker and 29 differentially expressed genes in expression profiles (Figure 2). TLR1 and TLR2 function together to recognize lipopeptides with a triacylated N-terminal cysteine. TLR1 is only mildly expressed in *T. nigroviridis *tissues and slightly upregulated in the spleens of LPS-injected fish [27]. Our data demonstrated that *TLR1 *was upregulated while *TLR2 *was downregulated at 24 h after *A. hydrophila *infection (Figure 5A). This result was partly consistent with that reported by Baoprasertkul et al., in which *TLR2 *expression in the spleens of channel and blue catfish was downregulated initially but upregulated 1 day postinfection with *Edwardsiella ictaluri *[28]. Bacterial infection has also been shown to induce *TLR3 *mRNA expression in zebrafish and channel catfish, as well as in channel-blue backcross hybrids following infection with *E. tarda *and *E. ictaluri *[25,29]. In our study, *TLR3 *expression was also upregulated 22.5-fold postinfection (Figure 5A), suggesting that this receptor might be involved in the immune response to bacterial infection in fish in addition to recognizing double-stranded RNA as in mammals. TLR22 is a fish-specific member of this family [30] that has also been found in the large yellow croaker. Recently, TLR22 was found located on the pufferfish cell surface recognizing long dsRNA sequences, whereas mammalian nucleic acid-sensing TLRs are localized in endosomes or the ER of myeloid cells, indicating that TLR22 may be a functional substitute for mammalian TLR3 that monitors for infections by double-stranded RNA viruses [25]. *TLR22 *was downregulated in the expression profile, implying that *TLR22 *was suppressed in the early period of *A. hydrophila *infection. Taken together, these results indicate that *TLRs *are regulated by various components of Gram-negative bacteria, suggesting that multiple TLR-mediated signaling cascades may simultaneously be involved in immune response to bacterial infection.

In our study, *A. hydrophila *infection led to a dramatic increase in the expression of proinflammatory cytokines such as *IL-1β*, *IL-8*, and *TNF-α *(Table 3). Studies have reported that these cytokines are induced within 24 h in human monocytes following Gram-positive and Gram-negative bacterial infection [31]. IL-1β is considered the prototypic multifunctional cytokine that affects nearly all cell types, either alone or in combination with other cytokines response to infection, injury, or immunologic challenge [32]. IL-8 is a proinflammatory CXC chemokine that has been shown to be regulated by a number of different stimuli including inflammatory signals (e.g., *TNF-α*, *IL-β*), chemical and environmental stresses, and steroid hormones [33]. Here, upregulation of these cytokines was observed by real-time PCR (Figure 5B), which is consistent with the observed findings in DeepSAGE. Therefore, the upregulation of these proinflammatory cytokines strongly suggests that the proinflammatory response may represent an important antibacterial mechanism at the early phase of infection.

The JAK-STAT pathway is initiated in response to cytokines, such as interleukins and IFNs, and growth factors present in the surrounding microenvironment [34]. Jak1 is a cytoplasmic tyrosine kinase that noncovalently associates with a variety of cytokine receptors and plays a nonredundant role in lymphoid cell precursor proliferation, survival, and differentiation [35,36]. STAT1, after activation by IFN-γ signaling, leads to the activation of peritoneal macrophages, resulting in enhanced bacteria killing and protection against lethal levels of *Listeria monocytogenes *infection in mice [23]. Genes encoding JAK-STAT pathway members, including *Jak1 *and *Stat1*, were found to be upregulated in our study (Table 3), suggesting that the JAK-STAT pathway may be affected by bacterial infection, which may result in changes in other cross-talk biological processes, such as NF-κB signaling pathway, TGF-β-activated SMAD pathway, and apoptosis [37].

Another signaling pathway affected by bacterial infection in the large yellow croaker was the MAPK cascade. This pathway has been demonstrated to regulate the expression of genes involved in the immune response to pathogens [38], cell differentiation, and cell death [39]. Modulation of MAPK activity in the common periwinkle in response to *Escherichia coli*-derived LPS has been studied [40]. Some key MAPK-related genes were identified in our transcriptome, including *Casp9*, *Rac1*, *Gadd45α*, and *Dusp7 *(Additional file 6, Table S6). Quantitative PCR analysis confirmed the differential expression of *Casp9 *and *Dusp7 *(Figure 5C). The Rho family GTPase Rac1 has been implicated in the control of the p38 MAPK signaling pathway by controlling β1 integrin. As shown in humans, dominant-negative Rac1 completely inhibits β1 integrin-induced p38 MAPK activation, whereas wild-type Rac1 overexpression causes a slight increase in β1 integrin-induced p38 MAPK activation [41]. Dual-specificity phosphatases including *Dusp7 *are a subset of protein tyrosine phosphatases, many of which dephosphorylate threonine and tyrosine residues on MAPKs and hence are also referred to as MAPK phosphatases (MKPs). The regulated expression and activity of DUSP family members in different cells and tissues control MAPK intensity and duration to determine the type of physiological response [42,43]. Therefore, the identified changes in gene expression in the large yellow croaker may facilitate the activation of the MAPK pathway and protect hosts against *A. hydrophila *infection.

Adaptive immunity is the process that leads to specific host resistance to infection [44]. T cells orchestrate responses against such foreign pathogens as viruses and bacteria. TCR and its downstream signaling cascades play a key role in these events. Here, we identified TCR pathway-related genes that were downregulated at 24 h after *A. hydrophila *infection. This complex process is shown in Figure 4, and genes expressed differentially are listed in Additional file 7, Table S7. *Lyn*, *Itk*, *Was*, *Ptpn6*, and *Jun *expression was downregulated, implying that the TCR signaling pathway may be suppressed in the early period (24 h) following bacterial infection. Studies have shown that a fine balance exists between a positive signal that initiates TCR cascade and a negative signal that controls the threshold, extent, and termination of TCR activation [45]. Several protein tyrosine phosphatases (PTPs) have been shown to function as negative regulators of the TCR signaling pathway by dephosphorylating activated signaling molecules [46,47]. Here, expression of *Ptpn6*, a member of the PTP family [48], was downregulated (Figure 5D), suggesting that although the TCR signaling pathway was suppressed by *A. hydrophila*, the host began to downregulate the expression of the PTPs to antagonize the repression. Clearly, there is a need for further studies to elucidate the precise roles of the PTP family members in the TCR signaling pathway in fish.

## Conclusions

Several recent studies have exploited novel high-throughput deep sequencing technology as a new method to advance further understanding of the mechanism of fish defense against infection [17]. We used the *A. hydrophila*-infected large yellow croaker as a model to study the immune response of fish to bacterial infection. Our analysis of the transcriptome and gene expression in *A. hydrophila*-infected large yellow croaker revealed changes in multiple signaling pathways involved in immunity in the large yellow croaker. The multiple TLR-mediated signaling cascades may be involved in early response to bacterial infection, causing the production of proinflammatory cytokines, chemokines, and other cytokines, which may result in the inflammatory response and affect other signal pathways such as JAK-STAT and MAPK. However, the TCR signaling pathway, a pivotal process in cellular immunity, was suppressed in the early period of *A. hydrophila *infection. The immune-related genes and signaling pathways involved in bacterial infection were identified and thereby provided valuable leads for further investigations into the immune response of fish.

## Methods

### Fish and infection experiments

Large yellow croakers (mean weight, 200 g) were purchased from a mariculture farm in Lianjian, Fuzhou, China. The fish were maintained at 25°C in aerated water tanks with a flow-through seawater supply. After 7 days of acclimation, these fish were used for the infection experiments. Twenty fish were injected intramuscularly with *A. hydrophila *at a dose of 1 × 10^8 ^cfu/200 g (This dose was chosen based on previous unpublished data) of fish. The strain of *A. hydrophila *(PPD 134/91) used in our manuscript was kindly provided by professor Xuanxian Peng [49]. A second group of 20 fish was injected with sterilized 0.9% NaCl at a dose of 0.2 ml/200 g of fish as a control [50]. The spleen tissues sampled at 12 h after infection with *A. hydrophila *were used for transcriptome analysis. The spleen tissues sampled at 24 h after injections with *A. hydrophila *or 0.9% NaCl were used for gene expression profiling analysis. All experiments were conducted in Third Institute of Oceanography, SOA, China. The protocols used meet the "Regulations for the Administration of Affairs Concerning Experimental Animals" established by the Fujian Provincial Department of Science and Technology on the Use and Care of Animals.

### RNA isolation

Total RNA was extracted from 50 to 100 mg of tissue with TRIZOL^® ^Reagent (Invitrogen, Carlsbad, CA, USA) according to the manufacturer's instructions. The RNA samples were incubated for 30 min at 37°C with 10 units of DNaseI (Takara, Dalian, China) to remove residual genomic DNA. The quality and quantity of the purified RNA were determined by measuring the absorbance at 260 nm/280 nm (A260/A280) using a Nanodrop^® ^ND-1000 spectrophotometer (LabTech, Holliston, MA, USA). The samples had an average RIN value of 8.9 according to Labon-chip analysis using the 2100 Bioanalyzer (Agilent Technologies, Santa Clara, CA, USA).

### Library preparation and sequencing

First, to survey the gene expression profile in the large yellow croaker and obtain longer transcript sequences for better annotation of the transcriptome, we constructed the entire library using the Mate Pair Library Preparation Kit. Then, to investigate the dynamics of gene expression after infection with *A. hydrophila*, we performed two tag-library preparations using the DeepSAGE: Tag Profiling for Nla III Sample Prep Kit from Illumina according to the manufacturer's instructions.

To better assemble the entire transcriptome *de novo*, a paired-end sequencing strategy was used for sequencing. A fragment sequencing strategy was used to sequence the tags. The data has been submitted to NCBI, and the accession number is SRA010789.13.

### Assembly of transcripts and annotation

Transcripts were assembled using the SOAP *de novo *software http://soap.genomics.org.cn/soapdenovo.html. As a result, 26,313 scaffolds were generated. To annotate these scaffolds, we first aligned them by using the zebrafish RefSeq mRNA database. The remaining non-annotated scaffolds were further aligned to the nr database. The annotated scaffolds were clustered and designated as unigenes when two or more query sequences were annotated to the same gene. The assembled contigs were used as a reference for annotating the DeepSAGE tags. GO and KEGG gene function were performed using DAVID [19].

### Identification of differentially expressed genes

Gene expression was measured by counting tags from normal and bacteria-infected fish and normalized to the total high-quality reads. High-throughput sequencing was performed using the Solexa/Illumina Genome Analyzer. To investigate differences in gene expression profiles, we analyzed genes between both libraries using the IDEG6 modeling methods [51]. GenMAPP 2.0 was used to show differences in expression in the different pathways [52].

### Quantitative real-time PCR

Quantitative real-time PCR was performed using the ABI Prism 7500 Detection System (Applied Biosystems, Foster City, CA, USA) with SYBR Green as the fluorescent dye according to the manufacturer's protocol (Takara). First-strand cDNA was synthesized from 2 μg of total RNA as described above and used as a template for real-time PCR with specific primers (Additional file 8, Table S8). Real-time PCR was performed in a total volume of 20 μl, and cycling conditions were 95°C for 5 min, followed by 40 cycles of 94°C for 5 s, 55°C for 20 s, and 72°C for 20 s. All reactions were performed in biological triplicates, and the results were expressed relative to the expression levels of β-actin in each sample by using the 2ΔΔCT method [53]. Each sample was first normalized for the amount of template added by comparison with the abundance of β-actin mRNA [54].

## Authors' contributions

JQA, SNH, and XHC participated in designing the research and helped write the manuscript. YNM performed the research. FD and PC analyzed the data and designed the tables and figures. YNM and FD wrote the manuscript. All authors read and approved the final manuscript.

## Supplementary Material

Additional file 1 **Table S1: Details on 8216 unigenes identified in the transcriptome of the large yellow croaker**. Click here for file

Additional file 2 **Table S2: Solexa tag libraries of the infected and normal large yellow croaker**. Click here for file

Additional file 3 **Table S3: Tags found to match sequences in the transcriptome**. Click here for file

Additional file 4 **Table S4: Details on 1996 differentially expressed genes in expression profile of large yellow croaker**. The data show the 1996 unigenes that were differentially expressed in the infected and normal large yellow croaker. The tag number, fold change, and P value are shown in the table. Click here for file

Additional file 5 **Table S5: GO function annotation results of 1996 differentially expressed genes using DAVID**. Gene Ontology was performed using DAVID. Click here for file

Additional file 6 **Table S6: Significant differentially expressed genes in MAPK signaling pathway**. Click here for file

Additional file 7 **Table S7: Significant differentially expressed genes in T cell receptor signaling pathway**. Click here for file

Additional file 8 **Table S8: Primers for relative quantitative real-time PCR**. Primers were designed from the sequences of the large yellow croaker transcriptome library by using Primer Premier 5.0. Click here for file
