# Supplementary material for: Transcriptome and expression profiling analysis revealed changes of multiple signaling pathways involved in immunity in the large yellow croaker during Aeromonas hydrophila infection
Source: BMC Genomics. 2010 Sep 22;11:506. doi: 10.1186/1471-2164-11-506 (PMC2997002; doi:10.1186/1471-2164-11-506)
Supplement: Additional file 2 — Table S2: Solexa tag libraries of the infected and normal large yellow croaker. [file 1471-2164-11-506-S2.DOC]

**Table S2: Solexa tag libraries of the infected and normal** large yellow croaker

|  | Infected fish | | Normal fish | |
| --- | --- | --- | --- | --- |
|  | Distinct Tag | Total Tag | Distinct Tag | Total Tag |
| Raw Data | 280663 | 5021994 | 294350 | 5581535 |
| Low Quality Tag | 1194 | 1205 | 785 | 793 |
| Adaptor Tag | 1 | 26 | 1 | 35 |
| Tag CopyNum =1 | 179361 | 179361 | 184992 | 184992 |
| Clean Tag | 100107 | 4841402 | 108572 | 5395715 |
| CopyNum >1 | 100107 | 4841402 | 108572 | 5395715 |
| CopyNum >5 | 37152 | 4662800 | 38822 | 5201753 |
| CopyNum >10 | 24207 | 4565444 | 26020 | 5105438 |
| CopyNum >20 | 16151 | 4447620 | 17816 | 4985312 |
| CopyNum >50 | 8883 | 4212312 | 9867 | 4728438 |
| CopyNum >100 | 5185 | 3948769 | 5656 | 4429275 |
