# Supplementary material for: Transcriptome and expression profiling analysis revealed changes of multiple signaling pathways involved in immunity in the large yellow croaker during Aeromonas hydrophila infection
Source: BMC Genomics. 2010 Sep 22;11:506. doi: 10.1186/1471-2164-11-506 (PMC2997002; doi:10.1186/1471-2164-11-506)
Supplement: Additional file 3 — Table S3: Tags found to match sequences in the transcriptome. [file 1471-2164-11-506-S3.DOC]

**Table S3: Tags found to match sequences in the transcriptome**

|  | Infected | | Normal | |
| --- | --- | --- | --- | --- |
|  | Distinct Tag | Total Tag | Distinct Tag | Total Tag |
| Clean Tag | 100107 | 4841402 | 108572 | 5395715 |
| Perfect Match(Sense) |  |  |  |  |
| 1 tag->1 gene | 6092 | 985663 | 6070 | 1007526 |
| 1 tag->n gene | 622 | 382867 | 610 | 452201 |
| 1bp MisMatch(Sense) |  |  |  |  |
| 1 tag->1 gene | 7304 | 234840 | 8376 | 215488 |
| 1 tag->n gene | 1870 | 72697 | 2075 | 111035 |
| Perfect Match(AniSense^*^) |  |  |  |  |
| 1 tag->1 gene | 4891 | 597875 | 4928 | 662267 |
| 1 tag->n gene | 343 | 56978 | 325 | 60257 |
| 1bp MisMatch(AntiSense) |  |  |  |  |
| 1 tag->1 gene | 5900 | 129024 | 6909 | 140413 |
| 1 tag->n gene | 563 | 30152 | 666 | 24764 |
| All Tag Mapping to Gene | 27585 | 2490096 | 29959 | 2673951 |
| Unambiguous Tag Mapping to Gene | 24187 | 1947402 | 26283 | 2025694 |
| Unknown Tag | 72522 | 2351306 | 78613 | 2721764 |

^*^ indicates the direction of the tags aligned on the contigs.
