# Supplementary material for: Transcriptome and expression profiling analysis revealed changes of multiple signaling pathways involved in immunity in the large yellow croaker during Aeromonas hydrophila infection
Source: BMC Genomics. 2010 Sep 22;11:506. doi: 10.1186/1471-2164-11-506 (PMC2997002; doi:10.1186/1471-2164-11-506)
Supplement: Additional file 6 — Table S6: Significant differentially expressed genes in MAPK signaling pathway. [file 1471-2164-11-506-S6.DOC]

Table S6.Significant differentially expressed genes in MAPK signaling pathway

| Accession No. | Gene | Fold change | P value | Expression level |
| --- | --- | --- | --- | --- |
| NM_001007404 | *Casp9* | 5.8 | 0.000013 | up |
| NM_200978 | *Prkcb1* | 1.95 | 0.012735 | up |
| NM_213058 | *Hspa5* | 1.98 | 0.000000 | up |
| NM_200576 | *Gadd45a* | 1.7 | 0.000000 | up |
| NM_199774 | *Dusp7* | 8/0 | 0.001319 | up |
| NM_199771 | *Rac1* | 5/0 | 0.012469 | up |
| NM_001109712 | *Casp1* | 4/0 | 0.026366 | up |
| NM_207094 | *Map3k12* | 0/17 | 0.000010 | down |
| NM_213538 | *Crkl* | 0/5 | 0.021440 | down |
| NM_199987 | *Jun* | 0/5 | 0.021440 | down |
| NM_131439 | *Raf1* | 0/5 | 0.021440 | down |
