# Supplementary material for: Transcriptome and expression profiling analysis revealed changes of multiple signaling pathways involved in immunity in the large yellow croaker during Aeromonas hydrophila infection
Source: BMC Genomics. 2010 Sep 22;11:506. doi: 10.1186/1471-2164-11-506 (PMC2997002; doi:10.1186/1471-2164-11-506)
Supplement: Additional file 7 — Table S7: Significant differentially expressed genes in T cell receptor signaling pathway. [file 1471-2164-11-506-S7.DOC]

Table S7. Significant differentially expressed genes in T cell receptor signaling pathway

| Accession No. | Gene | Fold change | P value | Expression level |
| --- | --- | --- | --- | --- |
| NM_213235^*^ | *Khdrbs1* | 2.7 | 0.004616 | up |
| NM_200628 | *Skap2* | 2.5 | 0.024788 | up |
| NM_001110452 | *Vasp* | 2.3 | 0.000000 | up |
| NM_212822 | *Pik3r2* | 1.8 | 0.003753 | up |
| NM_131884 | *Cebpb* | 2.5 | 0.000001 | up |
| NM_001020589 | *Zap70* | 4/0 | 0.026366 | up |
| NM_001007330 | *Cbl* | 4/0 | 0.026366 | up |
| CAQ15295^*^ | *Was* | -3.3 | 0.017699 | down |
| NM_001004543 | *Lyn* | -2.9 | 0.005425 | down |
| NM_199960 | *Ptpn6* | -2.2 | 0.035469 | down |
| NM_131059 | *Ctnnb1* | -1.9 | 0.004898 | down |
| NM_131104 | *Itk* | 0/5 | 0.021440 | down |
| NM_213538 | *Crkl* | 0/5 | 0.021440 | down |
| NM_199987 | *Jun* | 0/5 | 0.021440 | down |
| NM_194411 | *Ripk2* | 0/4 | 0.040678 | down |

^*^ indicates the gene is not shown in the map. The absence of this gene in the map is due to homolog gene conversion between *Mus musculus* and *Danio rerio*.
