# Supplementary material for: Transcriptome and expression profiling analysis revealed changes of multiple signaling pathways involved in immunity in the large yellow croaker during Aeromonas hydrophila infection
Source: BMC Genomics. 2010 Sep 22;11:506. doi: 10.1186/1471-2164-11-506 (PMC2997002; doi:10.1186/1471-2164-11-506)
Supplement: Additional file 8 — Table S8: Primers for relative quantitative real-time PCR. Primers were designed from the sequences of the large yellow croaker transcriptome library by using Primer Premier 5.0. [file 1471-2164-11-506-S8.DOC]

Table S8. Primers for relative quantitative real-time PCR

| Gene name | Forward Primer(5’----3’) | Reverse Primer(5’----3’) |
| --- | --- | --- |
| β-actin | GACCTGACAGACTACCTCATG | AGTTGAAGGTGGTCTCGTGGA |
| *TLR1* | CTGTGCCACCGTTTGGATA | TTCGAGGTTGGGAATGAGG |
| *TLR2* | CTCTGAGAATTTCGTCCAGTCC | TGAGGCCACTCCAAGTAGGT |
| *TLR3* | AGCACCGACTTCATCTGCTTTG | TGGTCTTCCTGCTCGCATAGATG |
| *TLR22* | TTCTACAAAGTGAGTGGTGCT | ATCCTCCTCTTGAGGTTCC |
| *IL-1β* | GTGGAAAATTCAGCCAATC | TGAAGTTTCTGTGGCGTCT |
| *IL-8* | AGAATCTTCGTCGCCTCCA | ACCTCCTGGCCTGACACTT |
| *TNF-α* | TATGGCCGTAAGAACACCTA | GCAAACACACCGAAGAAAGT |
| *Zap70* | TGGAAACCACATGGAGTGT | CTTGGCCTTGTTCGATATG |
| *Casp9* | AGCGACACAAGAAGCTTCTG | CTGAAGCAGCTCCGCCAAAC |
| *Dusp7* | GAACTTCAGCTTCATGGGACA | GTGGTGATGGGACTGTGGA |
| *Ptpn6* | AAGTCGAGAAAGGGCGGAATA | GGCCAGCTCATGTACTGGTAGTG |
| *Vasp* | AGGTGAGGGCGACGGTCAT | AGTTTGGCGTGGCTTGGTT |
